# Producing Novel Fibrinolytic Isoindolinone Derivatives in Marine Fungus *Stachybotrys longispora* FG216 by the Rational Supply of Amino Compounds According to Its Biosynthesis Pathway

**DOI:** 10.3390/md15070214

**Published:** 2017-07-05

**Authors:** Ying Yin, Qiang Fu, Wenhui Wu, Menghao Cai, Xiangshan Zhou, Yuanxing Zhang

**Affiliations:** 1 State Key Laboratory of Bioreactor Engineering, East China University of Science and Technology, 130 Meilong Road, Shanghai 200237, China; yingy@sibs.ac.cn (Y.Y.); cmh022199@ecust.edu.cn (M.C.); xszhou@ecust.edu.cn (X.Z.); 2 College of Food Biotechnology, Shanghai Ocean University, 999 Huchenghuan Road, Shanghai 201306, China; m140208432@st.shou.edu.cn (Q.F.); whwu@shou.edu.cn (W.W.); 3 Shanghai Collaborative Innovation Center for Biomanufacturing, 130 Meilong Road, Shanghai 200237, China; 4 Institute of Plant Physiology and Ecology, Chinese Academy of Sciences, 300 Feng Lin Road, Shanghai 200032, China

**Keywords:** *Stachybotrys*, isoindolinone biosynthesis, genome mining, amino compound, fibrinolytic activity

## Abstract

Many fungi in the *Stachybotrys* genus can produce various isoindolinone derivatives. These compounds are formed by a spontaneous reaction between a phthalic aldehyde precursor and an ammonium ion or amino compounds. In this study, we suggested the isoindolinone biosynthetic gene cluster in *Stachybotrys* by genome mining based on three reported core genes. Remarkably, there is an additional nitrate reductase (NR) gene copy in the proposed cluster. NR is the rate-limiting enzyme of nitrate reduction. Accordingly, this cluster was speculated to play a role in the balance of ammonium ion concentration in *Stachybotrys*. Ammonium ions can be replaced by different amino compounds to create structural diversity in the biosynthetic process of isoindolinone. We tested a rational supply of amino compounds ((±)-3-amino-2-piperidinone, glycine, and l-threonine) in the culture of an isoindolinone high-producing marine fungus, *Stachybotrys longispora* FG216. As a result, we obtained four new kinds of isoindolinone derivatives (FGFC4–GFC7) by this method. Furthermore, high yields of FGFC4–FGFC7 confirmed the outstanding production capacity of FG216. Among the four new isoindolinone derivatives, FGFC6 and FGFC7 showed promising fibrinolytic activities. The knowledge of biosynthesis pathways may be an important attribute for the discovery of novel bioactive marine natural products.

## 1. Introduction

“Supply problem” is a major bottleneck in the discovery of marine drugs [1], and many research efforts on marine natural products (MNPs) are currently directed towards microorganisms. Marine microorganisms became the main source of novel MNPs (677 out of 1378) in 2014. Among them, marine fungi are the most talented MNP producers (426 out of 677) [2]. Considering their great ability to produce new compounds, marine fungi should be investigated more carefully. For instance, an increasing amount of information on fungi genomes is now available, which facilitates the discovery of biosynthesis pathways by genome mining. The knowledge of biosynthesis pathways is beneficial for MNP production.

**Figure 1:**
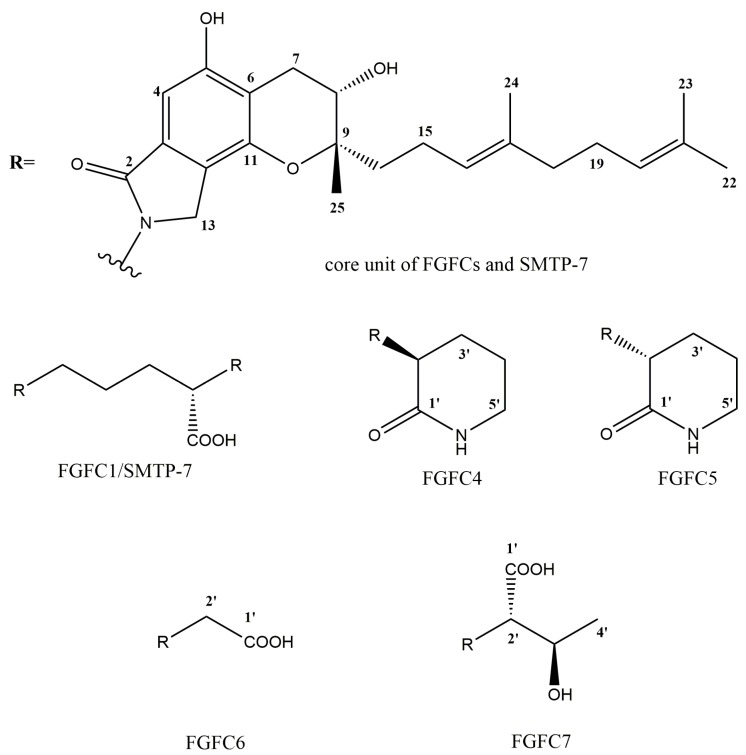
The structures of FGFCs and SMTP-7.

Since a high fat and high calorie diet has been a part of modern life, stroke becomes a serious health risk for humans, especially when they get old. The plasminogen/plasmin system plays a key role in thrombolysis [3]. Therefore, small-molecule modulators of plasminogen activation have attracted increasing attention in cardiovascular drug development [4]. Many isoindolinone derivatives isolated from *Stachybotrys microspora* IFO 30018 (known as *Stachybotrys microspora* triprenyl phenols, SMTPs) are plasminogen activators [5,6,7,8]. They share a common triprenyl isoindolinone core unit. Among them, SMTP-7 (see Figure 1) has shown the best fibrinolytic effect [7,9]. Most attractively, it was effective in treating thrombotic stroke in primates [10]. Marine fungus *S. longispora* FG216 can produce fungi fibrinolytic compound 1 (FGFC1), which has the same structure as SMTP-7 [11]. In addition, the production capacity of FG216 is outstanding. It has been shown to produce FGFC1 on a 10 g/L level under an optimized glucose/ornithine replenishment strategy [12]. FGFC1 has also shown positive effects on thrombolysis and hemorrhagic activities, both in vitro and in vivo [13]. Thus, FG216 has the potential for manufacturing cardiovascular drugs.

The precursor of all SMTPs has been reported to be pre-SMTP, which is a kind of prenylated phthalic aldehyde, and pre-SMTP is derived from a meroterpenoid ilicicolin B (also called LL-Z1272β) [14]. Recently, three genes responsible for the biosynthesis of ilicicolin B have been discovered in *S. bisbyi* [15]. In this study, we tried to ascertain the complete biosynthetic gene cluster for FGFCs or SMTPs by genome mining based on the three reported core genes. According to the biosynthesis pathway, a rational supply strategy of amino compounds was developed for the production of other novel isoindolinone derivatives. Since FGFC1 is a high yield compound, abundant new derivatives could also be easily obtained. Additionally, their fibrinolytic activities were also tested to evaluate the potential of FG216 for manufacturing cardiovascular drugs more comprehensively.

## 2. Results and Discussion

### 2.1. Isoindolinone Biosynthesis Pathway in Stachybotrys

As mentioned above, the biosynthesis pathway of ilicicolin B, the common precursor of all isoindolinone derivatives in *Stachybotrys*, has recently been discovered in *S. bisbyi* PYH05-7 [15]. A non-reducing polyketide synthase (NR-PKS) StbA, a putative UbiA-like prenyltransferase (PT) StbC, and a non-ribosomal peptide synthase (NRPS)-like enzyme StbB were reported to be successively involved. Although the genome information of *S. bisbyi* was not available, homologous genes of *stbA-C* could be found in the genomes of *S. chartarum* and *S. chlorohalonata* [15,16]. Therefore, we tried to identify the complete gene cluster by genome mining. 

**Figure 2:**
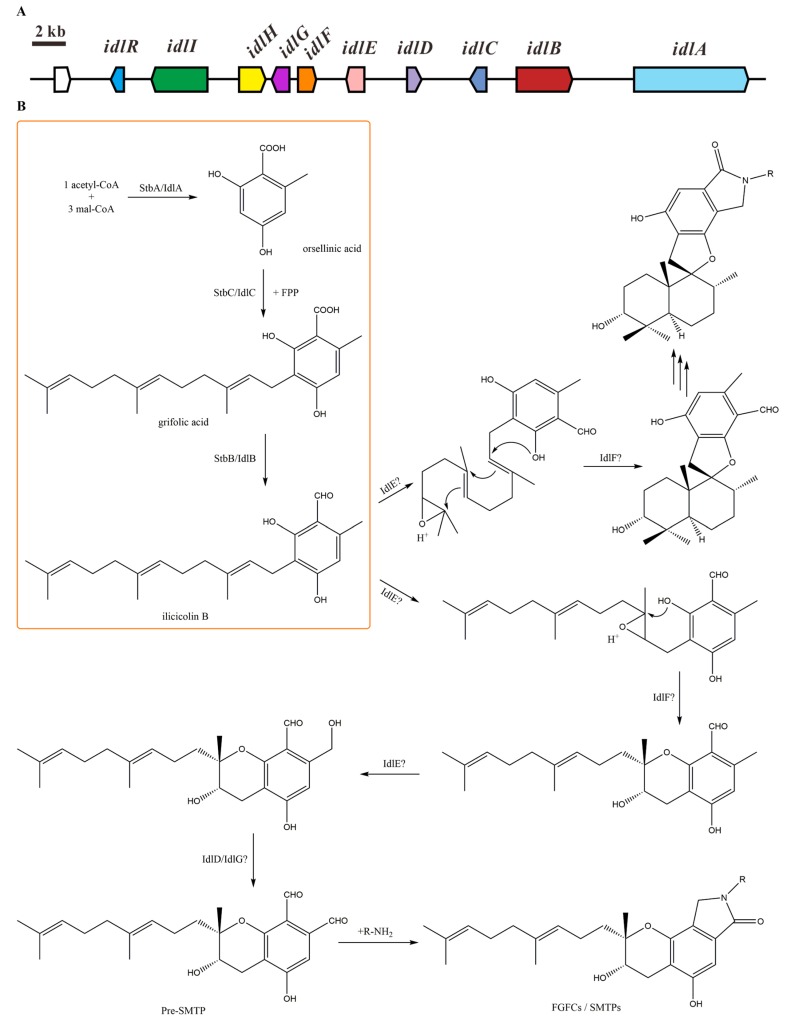
Proposed gene cluster and biosynthesis processes of isoindolinone derivatives in *Stachybotrys*. (**A**) Predicted gene cluster responsible for isoindolinone biosynthesis in *S. chlorohalonata* IBT 40285. (**B**) Proposed biosynthesis processes of isoindolinone derivatives in *Stachybotrys*. The three steps catalyzed by the three core genes are shown in an orange block.

As a result, we found a cluster containing 10 genes in their genomes (see Table 1). The gene cluster in *S. chlorohalonata* IBT 40285 was named as *idlA-I, R*. Nine genes in this cluster showed a high protein identity (above 94%) to their homologues in *S. chartarum* IBT 7711. The identity between *idlA-C* and *stbA-C* was a bit lower, at about 75%. The *idl* gene cluster and the proposed biosynthesis processes of isoindolinone derivatives in *Stachybotrys* are shown in Figure 2. The three steps catalyzed by the three core genes are shown in an orange block. Orsellinic acid is synthesized by NR-PKS StbA/IdlA in the first step. Then, a molecule of farnesyl pyrophosphate (FPP) is transferred by PT StbC/IdlC to form grifolic acid. The carboxyl group in grifolic acid is reduced by the reducing (R) domain of the NRPS-like enzyme StbB/IdlB [15]. According to the structures of the reported isoindolinone derivatives in *Stachybotrys*, we predict that two modes of cyclization occur afterwards, which may result from epoxidation on different double bonds of the farnesyl group. For example, stachybotrins [17,18] and chartarutines [19] share the same cyclization mode with FGFC1 and SMTPs, while stachybotrylactams [20], spirodihydrobenzofuranlactams [21], phenylspirodrimanes [22], spirocyclic drimanes [23], and chartarlactams [24] share the other mode. No matter which mode of cyclization occurs, the methyl group of orsellinic acid will be oxidized to an aldehyde group. The phthalic aldehyde precursor then combines with an ammonium ion or amino compounds to form different isoindolinone derivatives.

Homologous genes of NR-PKS, NRPS-like enzyme, and two putative short-chain dehydrogenases can be found in the gene cluster responsible for cichorine biosynthesis in the well-studied fungus *Aspergillus nidulans* [25,26], confirming their involvement in the biosynthesis of an isoindolinone skeleton. There is no prenylated structure in cichorine, so there are no homologues of the PT gene in this cluster. On the other hand, the homologous gene of PT can be found in another cluster responsible for the biosynthesis of meroterpenoids austinol and dehydroaustinol in *A. nidulans* [27]. A similar gene can also be found in the terretonin cluster in *A. terreus* [28]. These genes are related to the formation of the prenylated structure.

There is a nitrate reductase (NR) gene in the cluster of *S. chlorohalonata* IBT 40285, while its homologue in the cluster of *S. chartarum* IBT 7711 lacks a molybdopterin binding domain. Futhermore, there is a carboxylesterase gene in the cluster of IBT 7711 that does not occur in IBT 40285. We speculated that mutations might occur in this locus in the evolution process of *S. chartarum*. On the other hand, there is another intact NR gene (not in this cluster) in both genomes of IBT 40285 and IBT 7711. This suggests that there is or used to be an additional copy of the NR gene in the isoindolinone biosynthesis cluster in *Stachybotrys*. 

An additional gene is probably a resistance gene. In the case of *A. terreus*, there is an additional copy of the 3-hydroxy-3-methylglutaryl-coenzyme A (HMG-CoA) reductase gene in the lovastatin (HMG-CoA reductase inhibitor) cluster, and there is an additional copy of the ATP synthase β-chain gene in the citreoviridin (ATP synthase *β*-chain inhibitor) cluster [29]. As we mentioned, an isoindolinone skeleton is formed by the reaction between the phthalic aldehyde precursor produced by the cluster and ammonium ion or amino compounds. This reaction is reported to happen spontaneously [14], so ammonium ions will be wiped off by the product of this cluster. In contrast, NR is the rate-limiting enzyme of nitrate reduction, which is an important step in the formation of ammonium. Consequently, we speculated that this cluster might play a role in the balance of ammonium ion concentration in *Stachybotrys*.

### 2.2. Production of New FGFCs by Rational Amino Compounds Supply

To generate novel isoindolinone derivatives, the spontaneous reaction between the phthalic aldehyde and an ammonim ion can be manipulated by substituting various amino compounds. In other words, it is likely to control the structures of the isoindolinone derivatives by the supply of amino compounds. In the study of SMTPs, amino acids [6], glucosamine, sulfanilic acid [7], phenylamine [8], phenylglycine [30], and naphthenic amine [31] were used as amino precursors. Likewise, a high level production of FGFC1 was achieved in FG216 by a sufficient l-ornithine supply [12]. So novel isoindolinone derivatives were expected to be obtained through the rational supply of amino compounds. In this study, we tested four amino compound precursors (3-amino-2-piperidinone, (3*S*)-3-amino-2-piperidinone hydrochloride, glycine, and l-threonine). 3-Amino-2-piperidinone is the lactone of l-ornithine. Glycine and l-threonine are amino acids. They are all analogues of l-ornithine, so the isoindolinone derivatives derived from them are comparable with FGFC1 in fibrinolytic activities.

**Figure 3:**
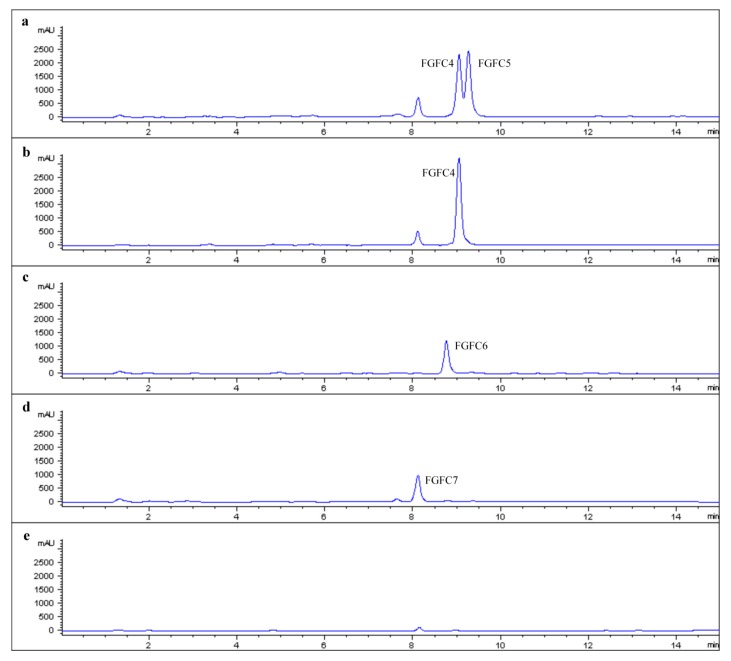
HPLC assay of *S. longispora* FG216 metabolites with different amino compounds supply. ((**a**) 3-amino-2-piperidinone, (**b**) (3*S*)-3-amino-2-piperidinone hydrochloride, (**c**) glycine, (**d**) l-threonine, and (**e**) no supply control.)

The fermentation medium used in this study was the optimized medium for FGFC1 production [12] without an l-ornithine hydrochloride supply. The meroterpenoid precursor for FGFCs could be produced on a high level in this medium, and new isoindolinone derivatives could be easily detected when other amino compounds were supplied. As shown in Figure 3, obvious new product peaks can be observed compared with the no supply control (Figure 3e).

3-Amino-2-piperidinone is a racemic mixture, so two adjacent peaks occur in Figure 3a. FGFC4 was recognized as the product of (3*S*)-3-amino-2-piperidinone, and FGFC5 was recognized as the product of (3*R*)-3-amino-2-piperidinone by comparing it with the optical product derived by (3*S*)-3-amino-2-piperidinone hydrochloride supply (Figure 3b). Considering the possible toxicity, 3-amino-2-piperidinone and its hydrochloride were only fed on 0.2% concentration. Consequently, we collected a 238 mg mixture of FGFC4 and FGFC5 (feeding 3-amino-2-piperidinone) and 163 mg FGFC4 (feeding (3*S*)-3-amino-2-piperidinone hydrochloride) from 100 mL broth, respectively. In our former study, 913 mg FGFC1 could be derived from 100 mL broth, when 1.3% l-ornithine hydrochloride was fed. Therefore, the feeding concentration of 3-amino-2-piperidinone could be further increased to achieve a higher production under the premise of not affecting the growth of FG216. Some mixture (FGFC4 and FGFC5) was further separated, and 40 mg FGFC5 was collected.

Glycine (Figure 3c) and l-threonine (Figure 3d) were fed on 0.5% concentration, but only 62 mg FGFC6 and 72 mg FGFC7 were derived from 100 mL broth, respectively. We predict that large amounts of these two amino acids are probably utilized for cell growth and energy metabolism, since they are essential amino acids. Therefore, the way to feed essential amino acids may need further modification, e.g., feeding in the beginning of the stable growth phase.

Overall, the gram level per liter production of FGFC4–FGFC7 derived by a non-optimized method was an inspiring start, which confirmed the outstanding production capacity of FG216.

### 2.3. Structure Determination of New FGFCs

**Figure 4:**
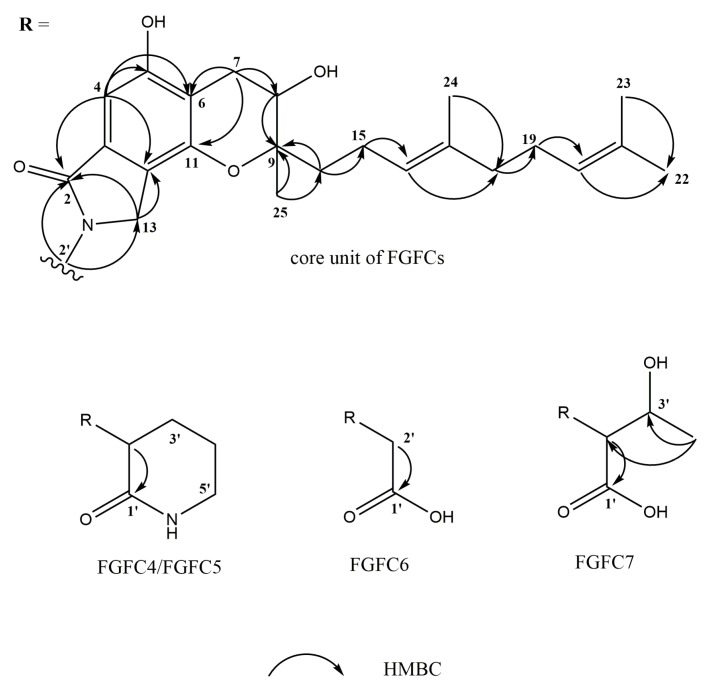
The HMBC correlation data of FGFC4–FGFC7.

FGFC4–FGFC7 all had the same UV absorption feature (λ_max_ 214, 258, 302 nm) as FGFC1, so we speculated that they had the same core unit as FGFC1 (Figure 1). As mentioned above, the *N*-linked side chains of FGFC4–FGFC7 were derived from (3*S/R*)-3-amino-2-piperidinone, glycine and l-threonine, respectively. Thus, we suggested the structures of FGFC4–FGFC7 in Figure 1. Then, we verified this speculation by ESI-TOF-MS and NMR. The NMR data of FGFC4–FGFC7 is shown in Table 2 and Figures S1–S14, and the HMBC correlations are shown in Figure 4. The NMR data also inferred that the four compounds shared a common core unit, which was also consistent with that of FGFC1 [11] or SMTP-7 [32].

The molecular formula of FGFC4 was determined as C_28_H_38_O_5_N_2_ according to the ESI-TOF-MS peaks at *m/z* 483.2813 [M + H]^+^ and the NMR data. Six aromatic singlets in the ^13^C NMR spectrum at δ_C_ 132.4 (C-3), 100.9 (C-4), 158.0 (C-5), 113.5 (C-6), 150.0 (C-11), and 121.9 (C-12), along with an aromatic singlet in the ^1^H NMR spectrum at δ_H_ 6.77 (1H, s, H-4), suggested the signal of a benzene ring, which was consistent with the UV absorption at 258 and 302 nm. HMBC interactions from H-4 to C-5, C-6, and C-11 also confirmed the existence of a benzene ring. Among these aromatic carbons, C-5 and C-11 were supposed to link with a hydroxy group or oxygen atom, according to their higher chemical shift. H-4 showed a correlation to a carbonyl carbon C-2 (δ_C_ 171.6). Two methylene protons, H-13-1 (δ_H_ 4.36, 1H, d) and H-13-2 (δ_H_ 4.24, 1H, d), showed correlations to C-2 and C-12. Furthermore, the methylene carbon C-13 (δ_C_ 46.8) was likely to link with the nitrogen atom because of its high chemical shift. Thus, an isoindolinone nucleus was recognized. Two methylene protons, H-7-1 (δ_H_ 3.00, 1H, dd) and H-7-2 (δ_H_ 2.67, 1H, dd), showed correlations to two aromatic carbons, C-6 and C-11, and a methylidyne carbon, C-8 (δ_C_ 68.3). The methylidyne proton H-8 (δ_H_ 3.90, 1H, dd) had a correlation to a quaternary carbon, C-9 (δ_C_ 80.2). Both C-8 and C-9 exhibited a high chemical shift, indicating that they linked with a hydroxy group or oxygen atom. According to the core unit structure of FGFC1, C-9 and C-11 were linked by an oxygen atom, constituting a pyran ring. Two methylene protons H_2_-14 (δ_H_ 1.69, 2H, m) and three methyl protons H_3_-25 (δ_H_ 1.29, 3H, s) showed correlations to the pyran ring at C-9. These two methylene protons H_2_-14 also showed correlations to a geranyl, which had an ^1^H NMR spectrum of δ_H_ 2.20 (2H, m, H_2_-15), 5.16 (1H, t, H-16), 1.99 (2H, m, H_2_-18), 2.07 (2H, m, H_2_-19), 5.10 (1H, t, H-20), 1.67 (3H, s, H_3_-22), 1.59 (3H, s, H_3_-23), and 1.60 (3H, s, H_3_-24), and an ^13^C NMR spectrum of δ_C_ 22.6 (C-15), 125.5 (C-16), 136.3 (C-17), 40.8 (C-18), 27.7 (C-19), 125.4 (C-20), 132.2 (C-21), 25.9 (C-22), 17.8 (C-23), 16.0 (C-24). Hence, geranyl linked to the pyran ring through a methylene. As for the structure of the *N*-linked side chain, it showed a high consistency with 3-amino-2-piperidinone. The central methylidyne proton H-2’ (δ_H_ 4.80, 1H, dd) showed correlations to C-2 and C-13, confirming the linkage between the *N*-linked side chain and isoindolinone nucleus. It also showed a correlation to a carbonyl carbon, C-1’ (δ_C_ 171.7), which had a similar chemical shift to C-2. Among the three methylenes, C-5’ (δ_C_ 43.0) had the highest chemical shift, suggesting that it was the one linked with acylamino. FGFC5 showed the same molecular weight and NMR spectrum as FGFC4, which confirmed that they were chiral isomers.

The molecular formula of FGFC6 was determined as C_25_H_33_O_6_N according to the ESI-TOF-MS peak at *m/z* 442.2233 [M − H]^−^ and the NMR data. FGFC6 had the same core unit as FGFC4 and FGFC5. The structure of its *N*-linked side chain was very simple, since the amino compound precursor was glycine. The methylene protons H_2_-2’ (δ_H_ 4.34, 2H, s) showed correlations to C-2, C-13, and carboxyl carbon C-1’ (δ_C_ 171.7).

The molecular formula of FGFC7 was determined as C_27_H_37_O_7_N according to the ESI-TOF-MS peak at *m/z* 486.2544 [M − H]^−^ and the NMR data. FGFC7 also had the same isoindolinone core unit. Its *N*-linked side chain was derived from l-threonine. The central methylidyne proton H-2’ (δ_H_ 4.89, 1H, d) also showed correlations to C-2, C-13, and C-1’ (δ_C_ 173.0). The methylidyne carbon C-3’ (δ_C_ 68.9) linked with a hydroxy group. The methyl protons H_3_-4’ (δ_H_ 1.20, 3H, d) showed correlations to both C-2’ (δ_C_ 61.4) and C-3’.

### 2.4. Fibrinolytic Activities of New FGFCs

The reciprocal activation of prourokinase (pro-uPA) and plasminogen (plg) is believed to play a key role in tissue fibrinolysis. Compared with urokinase-type plasminogen activator (uPA), pro-uPA has a weak intrinsic activity, which can convert plg into plasmin (plm) [33,34]. Plasmin then converts pro-uPA into activeuPA. Accordingly, more plg can be converted into plm by uPA. A fibrinolytic reaction occurs.

This reciprocal activation process can be sped up when more pro-uPA is supplied. In addition, a small molecule fibrinolytic compound can further promote the reciprocal activation of pro-uPA and plg. In this study, we tested the fibrinolytic activities of FGFC4–FGFC7. The rate of urokinase catalyzing chromogenic substrate S-2444 to form nitroaniline was set as a standard to measure the activation degree of the enzymatic reaction system. The relative reaction rate of the negative control (enzymatic reaction system with no isoindolinone derivatives or extra pro-uPA) in the first 50 min was set as 1. The folds of the reaction rate enhanced by positive controls (extra 30 nmol/L pro-uPA and 0.1 g/L FGFC1, which were the most suitable working concentrations for them respectively) and test samples (different concentrations of FGFC4–FGFC7) were determined as relative activities. 

**Figure 5:**
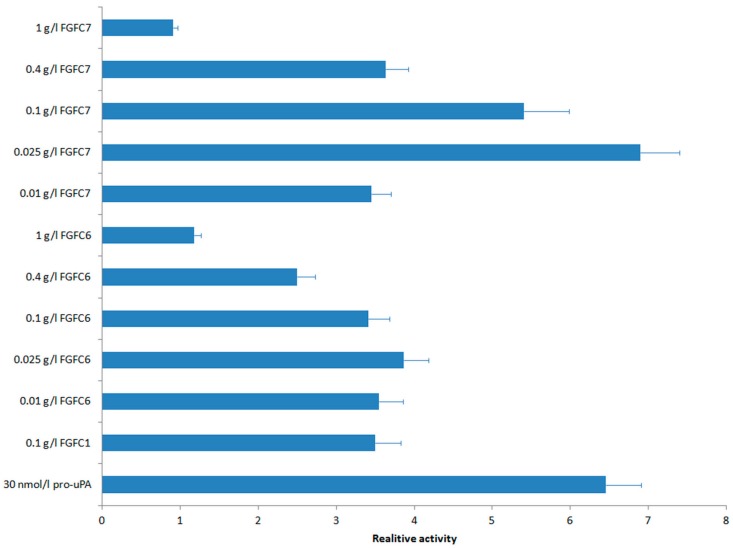
The relative activities of FGFC6 and FGFC7 in different concentrations. (Extra 30 nmol/L pro-uPA and 0.1 g/L FGFC1 were set as positive controls, respectively. The relative activity of no supply negative control was set as 1.)

It was a pity that the supplies of FGFC4 and FGFC5 did not promote the enzyme reaction rate. The relative activities of FGFC6 and FGFC7 under different concentrations are shown in Figure 5. The best working concentrations for FGFC6 and FGFC7 were both 0.025 g/L, which was much lower than that of FGFC1 (0.1 g/L), suggesting good application prospects for them. The relative activity of 0.025 g/L FGFC7 was 6.90, which was much higher than that of 0.1 g/L FGFC1 (3.50, *p* < 0.05), and roughly equivalent to that of extra 30 nmol/L pro-uPA (6.46). Additionally, the relative activity of 0.025 g/L FGFC6 was 3.86, which was roughly equivalent to that of 0.1 g/L FGFC1. Moreover, 0.1 g/L FGFC7 also showed a higher relative activity (5.41) than 0.1 g/L FGFC1 (*p* < 0.05). Some other concentrations (0.01 g/L FGFC6 and FGFC7, 0.1 g/L FGFC6 and 0.4 g/L FGFC7) showed similar relative activities to 0.1 g/L FGFC1. In contrast, high concentrations of FGFC6 and FGFC7 did not show good effects, especially 1 g/L FGFC6 and FGFC7, which did not exhibit a significant difference to the negative control.

Diversity of structure resulted in the diversity of bioactivity. Hasegawa et al. [7] summarized that negative ionization groups such as the carboxyl or sulfonic acid group in the *N*-linked side chain of SMTPs was a necessary basis for their plasminogen activation activity. Our results agreed with this conclusion. When ornithine was lactonized to 3-amino-2-piperidinone, the loss of the carboxyl acid group in the *N*-linked side chain resulted in inactive FGFC4 and FGFC5. However, FGFC6 and FGFC7, which were gained from amino acids, showed excellent activities that were even better than FGFC1, exhibiting potential application prospects. 

Besides plasminogen activation activity, isoindolinone derivatives also showed other important bioactivities. For example, many SMTPs showed anti-inflammatory activities, which were attributable to their abilities to inhibit soluble epoxide hydrolase (sEH) [31]. Stachybotrin C was a neuritogenic compound [35]. Chartarutines B, G, and H exhibited significant inhibitory effects against HIV-1 virus [18]. Stachybotrylactams were immunosuppressant [19]. Spirodihydrobenzofuranlactams showed antagonistic effects in the endothelin receptor binding assay [36]. Spirocyclic drimanes exhibited antibacterial activity against the clinically relevant methicillin-resistant *Staphylococcus aureus* (MRSA) [22]. Chartarlactams showed antihyperlipidemic effects in HepG2 cells [23]. Therefore, these new FGFCs will be detected for other bioactivities in the future in order to understand their application prospects more adequately.

In summary, we explored the isoindolinone biosynthesis pathway in *Stachybotrys*, and obtained four new isoindolinone derivatives (FGFC4–FGFC7) from marine fungus *S. longispora* FG216 by the rational supply of amino compounds. Enantiomers FGFC4 and FGFC5 were achieved by feeding racemic mixture 3-amino-2-piperidinone. However, they did not show fibrinolytic activities for their lack of negative ionization groups in the *N*-linked side chains. FGFC6 and FGFC7 were achieved by feeding glycine and l-threonine, respectively, and they showed competitive fibrinolytic activities. Especially, FGFC7 had a comparable effect to pro-uPA, although fermentation optimization was needed to improve the yield.

## 3. Materials and Methods

### 3.1. General Experimental Procedures

Extraction was performed on an ultrasonicator (KQ-800, Kunshan Ultrasonic Instruments Co., Ltd., Kunshan, China). High performance liquid chromatography (HPLC) was performed on an Agilent 1200 HPLC system (Agilent Technologies, Santa Clara, CA, USA) with a C18 column (ZORBAX Eclipse XDB, 150 mm × 4.6 mm, 5 μm, 100 Å-spherical silica) and a semipreparation C18 column (ZORBAX Eclipse XDB, 250 mm × 9.4 mm, 5 μm, 100 Å-spherical silica). The molecular weight was determined by the Agilent electrospray ionization time of flight mass spectrometry (ESI-TOF-MS) 6230. A nuclear magnetic resonance (NMR) assay was performed on Bruker AM-400 spectrometers (Bruker Daltonics Inc., Billerica, MA, USA). Fibrinolytic activity was detected by a microplate reader (SH-1000, CORONA, Ibarakiken, Japan).

### 3.2. Strain, Medium, and Cultural Conditions

*Stachybotrys longispora* FG216 (CCTCCM 2012272) was kindly provided by Shanghai Ocean University.

The sporiparous medium was potato sucrose agar (PSA), which contained 200 g/L potato, 20 g/L sucrose, and 20 g/L agar. The seed medium contained 35 g/L glucose, 10 g/L soluble starch, 20 g/L defatted soybean flour (Sigma, St. Louis, MO, USA), 5 g/L bacteriological peptone (Oxoid, Basingstoke, UK), 5 g/L beef extract paste (Oxoid, Basingstoke, UK), 3 g/L yeast extract (Oxoid, Basingstoke, UK), 2 g/L NaCl, 0.5 g/L K_2_HPO_4_, and 0.05 g/L MgSO_4_. The fermentation medium contained 125 g/L sucrose, 3.3 g/L NaNO_3_, 0.7 g/L yeast extract (Oxoid, Basingstoke, UK), 0.625 g/L KCl, 0.4 g/L MgSO_4_·7H_2_O, 0.07 g/L K_2_HPO_4_·3H_2_O, 18.75 mg/L FeSO_4_·7H_2_O, 6.5 mg/L CaCl_2_, and 3.125 mg/L CoCl_2_·6H_2_O. The seed and fermentation media were adjusted to pH 5.8 before sterilization. All media were sterilized at 121 °C for 20 min.

FG216 was statically incubated on PSA at 25 °C for 7 d to induce spore formation. Then, the spores were washed with sterile water, and 1 × 10^6^ spores were inoculated into 30 mL seed medium in a 250 mL Erlenmeyer flask and incubated at 25 °C, 180 rpm for 2.5 d to obtain the first-stage seed. Second-stage seed was gained by 1.5 mL of first-stage seed inoculated into 30 mL seed medium and incubated under the same conditions for 1 d. Finally, 1.5 mL second-stage seed was inoculated into 30 mL fermentation medium and incubated under the same conditions for 10 d.

### 3.3. Amino Compouds Supply

3-Amino-2-piperidinone was purchased from Ark Pharm, Inc. (Libertyville, IL, USA). (3*S*)-3-Amino-2-piperidinone hydrochloride was purchased from Shanghai Macklin Biochemical Co., Ltd. (Shanghai, China). Glycine and l-threonine were purchased from Shanghai Sangon Biotechnology Co., Ltd. (Shanghai, China). 

Each of them was dissolved in distilled water (3-amino-2-piperidinone in ethanol), sterilized with a millipore filter, and fed into the fermentation medium at a concentration of 0.2% (3-amino-2-piperidinone and (3*S*)-3-amino-2-piperidinone hydrochloride) or 0.5% (glycine and l-threonine) on day 0.

### 3.4. Metabolites Detection and Isolation

One hundred milliliter of fermentation broth was centrifuged at 5000× *g* for 20 min, and the precipitate was extracted with 100 mL methanol. The mixture was placed in an ultrasonicator at a frequency of 40 kHz and a nominal power of 800 W for 30 min. The extraction was repeated three times. The crude extract was obtained by concentration in vacuo.

The crude extract was detected by an HPLC system and then separated with a semipreparation column. The operating temperature was 40 °C, the flow rate was 1 mL/min and 4 mL/min for detection and separation, respectively, and UV detection was set at 258 nm. The mobile phase was composed of acetonitrile and 0.1% formic acid solution. For detection, the ratio acetonitrile increased from 10 to 100% in 15 min. For separation, the ratio of acetonitrile was set as 50%. FGFC4, FGFC5, FGFC6, and FGFC7 were collected at 12.3 min, 13.3 min, 10.2 min, and 16.0 min, respectively.

### 3.5. Bioactivity Assays

Single chain urokinase-type plasminogen activator (pro-uPA), plasminogen (plg), and bovine serum albumin (BSA) were purchased from Sigma-Aldrich Co. (St. Louis, MO, USA). Chromogenic substrate S-2444 of urokinase was purchased from HYPHEN BioMed Co. (Neuville-sur-Oise, France).

These reagents were respectively dissolved in Tris-HCl buffer solution (50 mmol/L Tris-HCl, 100 mmol/L NaCl, pH 7.4). Their concentrations were as follows: 20 nmol/L pro-uPA, 10 nmol/L plg, 10 g/L BSA, and 2 mmol/L S-2444. FGFC4 and FGFC5 were dissolved in methanol. FGFC6 and FGFC7 were dissolved in sodium bicarbonate solution (pH 7.5). Ten microlitre of each solution was mixed in a 96 hole round bottom plate to form a 50 μL enzymatic reaction system. The nitroaniline was formed at 37 °C for 150 min, and detected in a microplate reader at 405 nm.

FGFC4–FGFC7 were tested on five concentrations (0.01, 0.025, 0.1, 0.4, 1 g/L). An enzymatic reaction system with no isoindolinone derivatives was set as the negative control. Enzymatic reaction systems with an extra 30 nmol/L pro-uPA (the best concentration for pro-uPA) or 0.1 g/L FGFC1 (the best concentration for FGFC1) were set as positive controls, respectively. The assay was repeated three times.

## Figures and Tables

**Table 1:** Predicted gene cluster responsible for isoindolinone biosynthesis in *Stachybotrys*. The gene cluster in *S. chlorohalonata* IBT 40285 was compared to its homologues in *S. chartarum* IBT 7711 and *S. bisbyi* PYH05-7.

Gene	Locus Tag	*S. chartarum* IBT 7711 Homologue	Protein Identity (%)	*S. bisbyi* PYH05-7 Homologue	Protein Identity (%)	Putative Function
*idlR*	S40285_07604	S7711_05923	98			Transcriptional regulator
*idlI*	S40285_07605	S7711_05924	78 (34% coverage)			Nitrate reductase (partial in *S. chartarum* IBT 7711)
		S7711_05925				Carboxylesterase
*idlH*	S40285_07606	S7711_05926	97			Esterase
*idlG*	S40285_07607	S7711_05927	100			Short-chain dehydrogenase
*idlF*	S40285_07608	S7711_05928	94			Isomerase/epimerase
*idlE*	S40285_07609	S7711_05929	97			Copper dependent oxidase
*idlD*	S40285_07610	S7711_05930	96			Short-chain dehydrogenase
*idlC*	S40285_10521	S7711_10996	98	*stbC*	76	PT
*idlB*	S40285_07611	S7711_05931	98	*stbB*	73	NRPS-like
*idlA*	S40285_07612	S7711_05932	98	*stbA*	75	NR-PKS

**Table 2:** ^1^H (400 MHz) and ^13^C NMR (100 MHz) data for new FGFC congeners (in MeOH-*d*_4_, *J* in Hz).

	FGFC4		FGFC5		FGFC6		FGFC7	
*No.*	δ_C_	δ_H_	δ_C_	δ_H_	δ_C_	δ_H_	δ_C_	δ_H_
2	171.6, C		171.5, C		171.7, C		172.6, C	
3	132.4, C		132.4, C		132.2, C		131.7, C	
4	100.9, CH	6.77 s	101.0, CH	6.77 s	101.1, CH	6.78 s	101.0, CH	6.80 s
5	158.0, C		158.2, C		158.0, C		157.8, C	
6	113.5, C		113.6, C		113.6, C		113.6, C	
7	27.7, CH_2_	3.00 dd (17.6, 5.4)	27.8, CH_2_	3.01 dd (17.7, 5.5)	27.8, CH_2_	3.00 dd (17.7, 5.4)	27.8, CH_2_	3.01 dd (17.7, 5.3)
		2.67 dd (17.6, 7.1)		2.66 dd (17.7, 7.4)		2.68 dd (17.7, 7.0)		2.70 dd (17.7, 6.8)
8	68.3, CH	3.90 dd (7.0, 5.6)	68.5, CH	3.89 dd (7.2, 5.7)	68.4, CH	3.90 t (6.2)	68.4, CH	3.91 t (6.0)
9	80.2, C		80.2, C		80.3, C		80.2, C	
11	150.0, C		150.0, C		150.0, C		150.0, C	
12	121.9, C		121.9, C		122.2, C		123.1, C	
13	46.8, CH_2_	4.36 d (16.7)	46.9, CH_2_	4.34 d (16.7)	49.6, CH_2_	4.39 s	48.6, CH_2_	4.65 m
		4.24 d (16.7)		4.25 d (16.7)				
14	38.6, CH_2_	1.69 m	38.6, CH_2_	1.71 m	38.6, CH_2_	1.69 m	38.5, CH_2_	1.70 m
15	22.6, CH_2_	2.20 m	22.6, CH_2_	2.21 m	22.6, CH_2_	2.20 m	22.6, CH_2_	2.19 m
16	125.5, CH	5.16 t (6.8)	125.6, CH	5.17 t (6.7)	125.5, CH	5.16 t (6.9)	125.4, CH	5.16 t (6.9)
17	136.3, C		136.2, C		136.3, C		136.3, C	
18	40.8, CH_2_	1.99 m	40.9, CH_2_	1.99 m	40.8, CH_2_	1.98 m	40.8, CH_2_	1.97 m
19	27.7, CH_2_	2.07 m	27.7, CH_2_	2.07 m	27.7, CH_2_	2.07 m	27.7, CH_2_	2.07 m
20	125.4, CH	5.10 t (7.0)	125.4, CH	5.09 t (6.9)	125.4, CH	5.08 t (6.9)	125.4, CH	5.07 t (6.9)
21	132.2, C		132.2, C		132.2, C		132.2, C	
22	25.9, CH_3_	1.67 s	25.9, CH_3_	1.67 s	25.9, CH_3_	1.66 s	25.9, CH_3_	1.65 s
23	17.8, CH_3_	1.59 s	17.8, CH_3_	1.59 s	17.8, CH_3_	1.58 s	17.8, CH_3_	1.57 s
24	16.0, CH_3_	1.60 s	16.0, CH_3_	1.61 s	16.0, CH_3_	1.60 s	16.0, CH_3_	1.58 s
25	18.8, CH_3_	1.29 s	18.6, CH_3_	1.28 s	18.8, CH_3_	1.30 s	19.0, CH_3_	1.31 s
1’	171.7, C		171.8, C		171.7, C		173.0, C	
2’	53.6, CH	4.80 dd (10.6, 7.1)	53.6, CH	4.78 dd (10.6, 6.9)	45.0, CH_2_	4.34 s	61.4, CH	4.89 d (3.8)
3’	27.3, CH_2_	2.13 m	27.3, CH_2_	2.14 m			68.2, CH	4.62 m
4’	22.9, CH_2_	2.03 m	22.9, CH_2_	2.03 m			20.7, CH_3_	1.20 d (6.3)
5’	43.0, CH_2_	3.37 brs	43.0, CH_2_	3.37 brs				

## Supplementary Materials

The following are available online at www.mdpi.com/1660-3397/15/7/214/s1, Figure S1: ^1^H NMR Spectrum of FGFC4 in MeOH-*d*_4_, Figure S2: ^13^C NMR Spectrum of FGFC4 in MeOH-*d*_4_, Figure S3: HSQC Spectrum of FGFC4 in MeOH-*d*_4_, Figure S4: HMBC Spectrum of FGFC4 in MeOH-*d*_4_, Figure S5: ^1^H NMR Spectrum of FGFC5 in MeOH-*d*_4_, Figure S6: ^13^C NMR Spectrum of FGFC5 in MeOH-d4, Figure S7: ^1^H NMR Spectrum of FGFC6 in MeOH-*d*_4_, Figure S8: ^13^C NMR Spectrum of FGFC6 in MeOH-*d*_4_, Figure S9: HSQC Spectrum of FGFC6 in MeOH-*d*_4_, Figure S10: HMBC Spectrum of FGFC6 in MeOH-*d*_4_, Figure S11: ^1^H NMR Spectrum of FGFC7 in MeOH-*d*_4_, Figure S12: ^13^C NMR Spectrum of FGFC7 in MeOH-*d*_4_, Figure S13: HSQC Spectrum of FGFC7 in MeOH-*d*_4_, Figure S14: HMBC Spectrum of FGFC7 in MeOH-*d*_4_.
