# Supplementary material for: Producing Novel Fibrinolytic Isoindolinone Derivatives in Marine Fungus Stachybotrys longispora FG216 by the Rational Supply of Amino Compounds According to Its Biosynthesis Pathway
Source: Mar Drugs. 2017 Jul 5;15(7):214. doi: 10.3390/md15070214 (PMC5532656; doi:10.3390/md15070214)
Supplement: Supplementary file 1 [file marinedrugs-15-00214-s001.pdf]

# Supplementary Materials: Producing Novel Fibrinolytic Isoindolinone Derivatives in Marine Fungus *Stachybotrys longispora* FG216 by the Rational Supply of Amino Compounds According to Its Biosynthesis Pathway

**Ying Yin, Qiang Fu, Wenhui Wu, Menghao Cai, Xiangshan Zhou and Yuanxing Zhang**

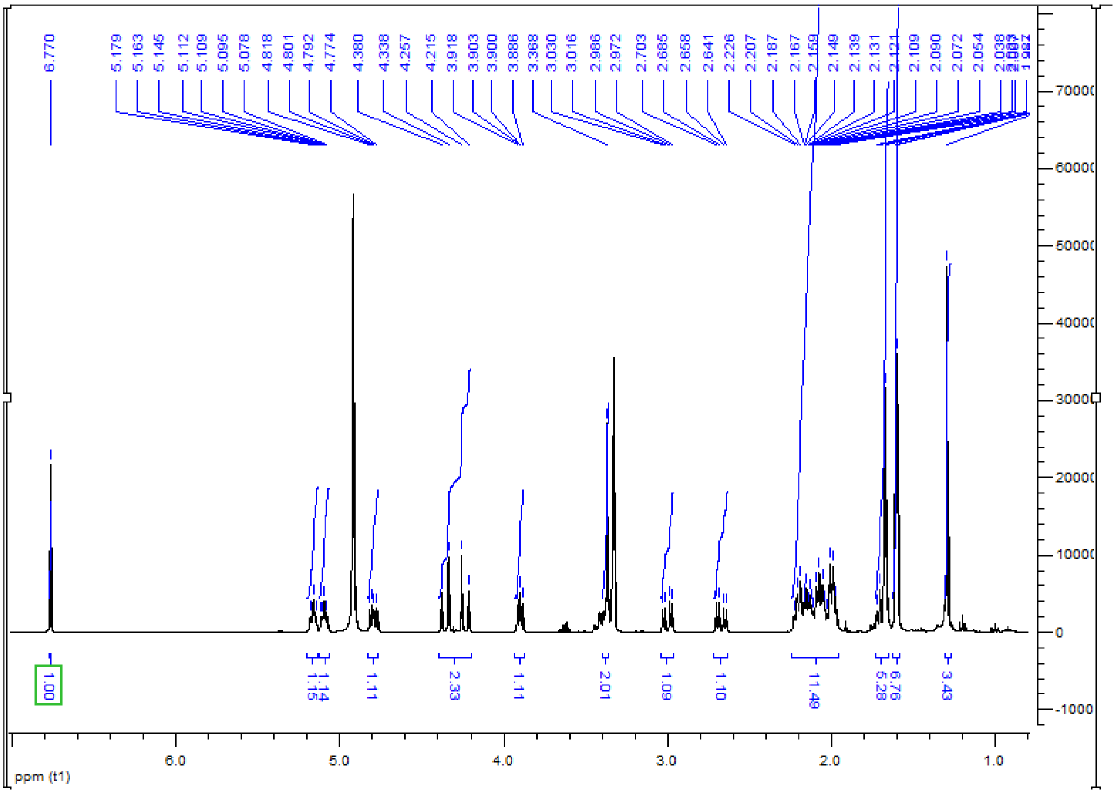


**Figure S1.** $^1$H NMR Spectrum of FGFC4 in MeOH-$d_4$.

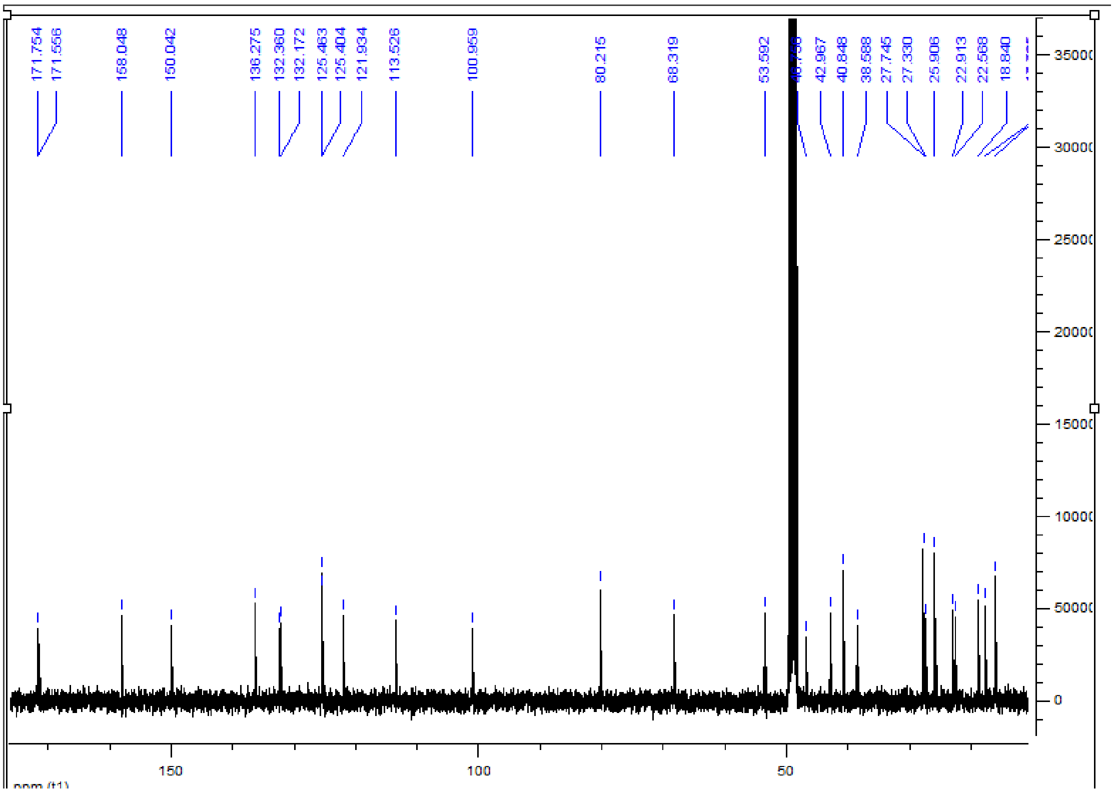


**Figure S2.** $^{13}$C NMR Spectrum of FGFC4 in MeOH-$d_4$.

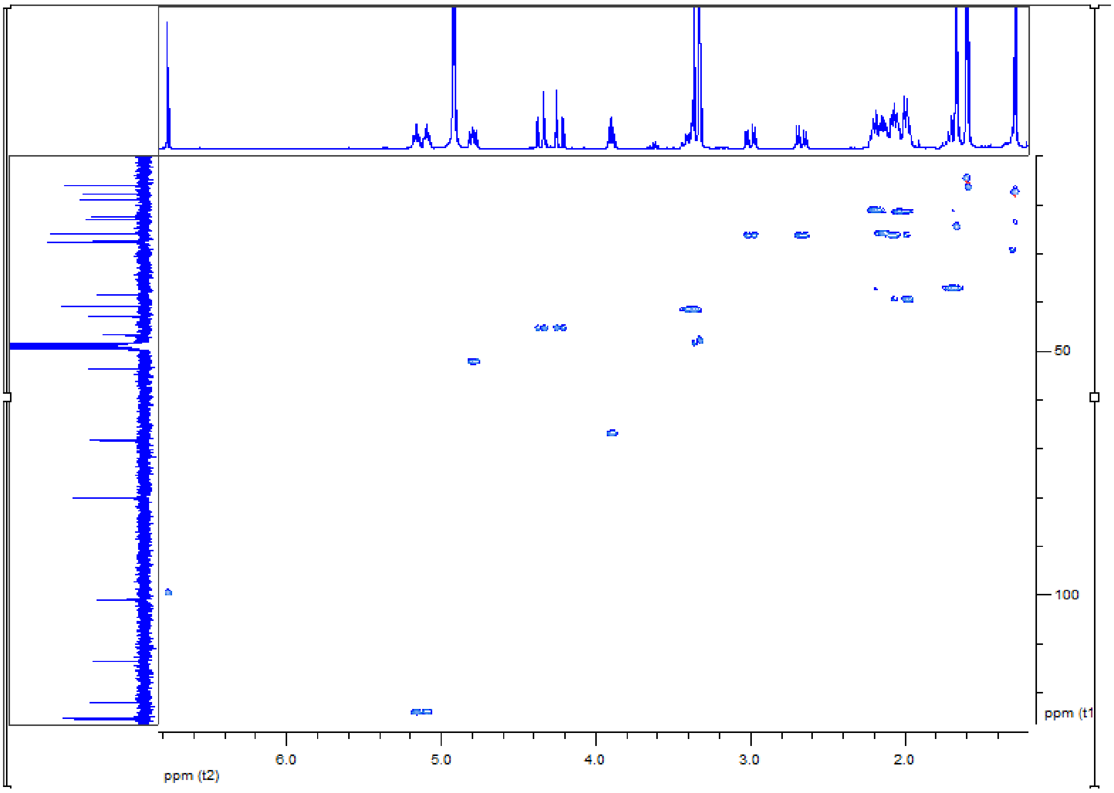


**Figure S3.** HSQC Spectrum of FGFC4 in MeOH-$d_4$.

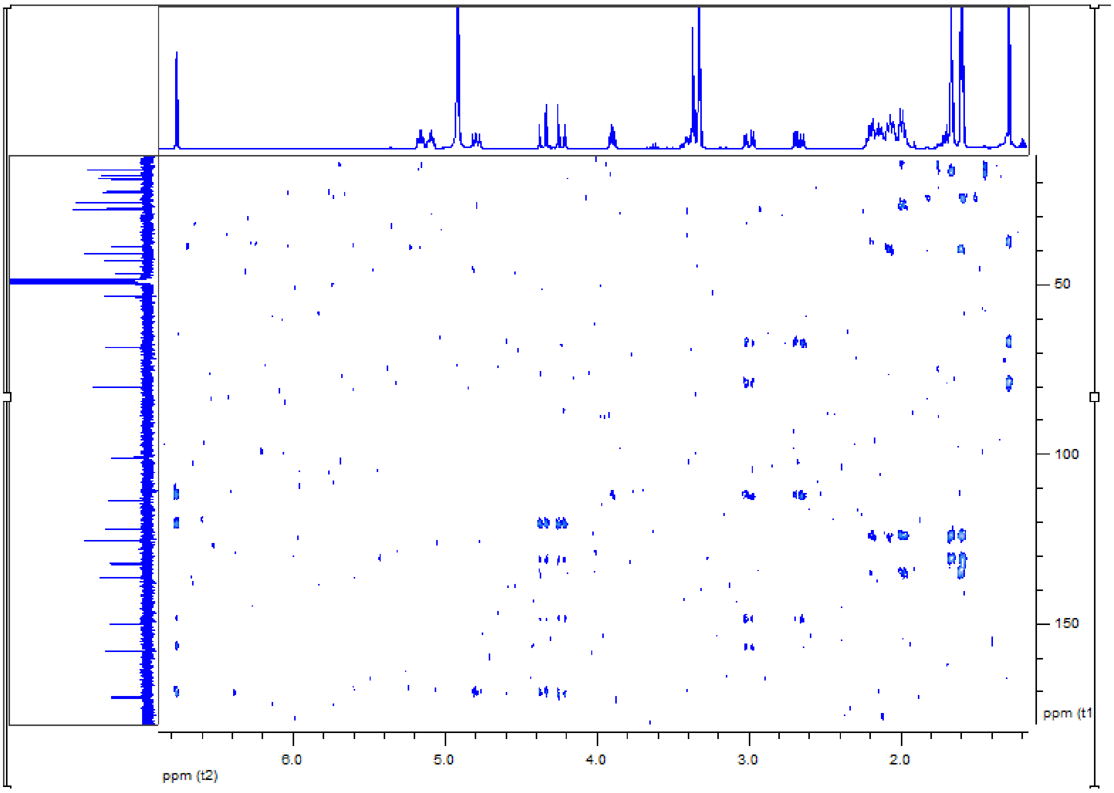


**Figure S4.** HMBC Spectrum of FGFC4 in MeOH-*d*4.

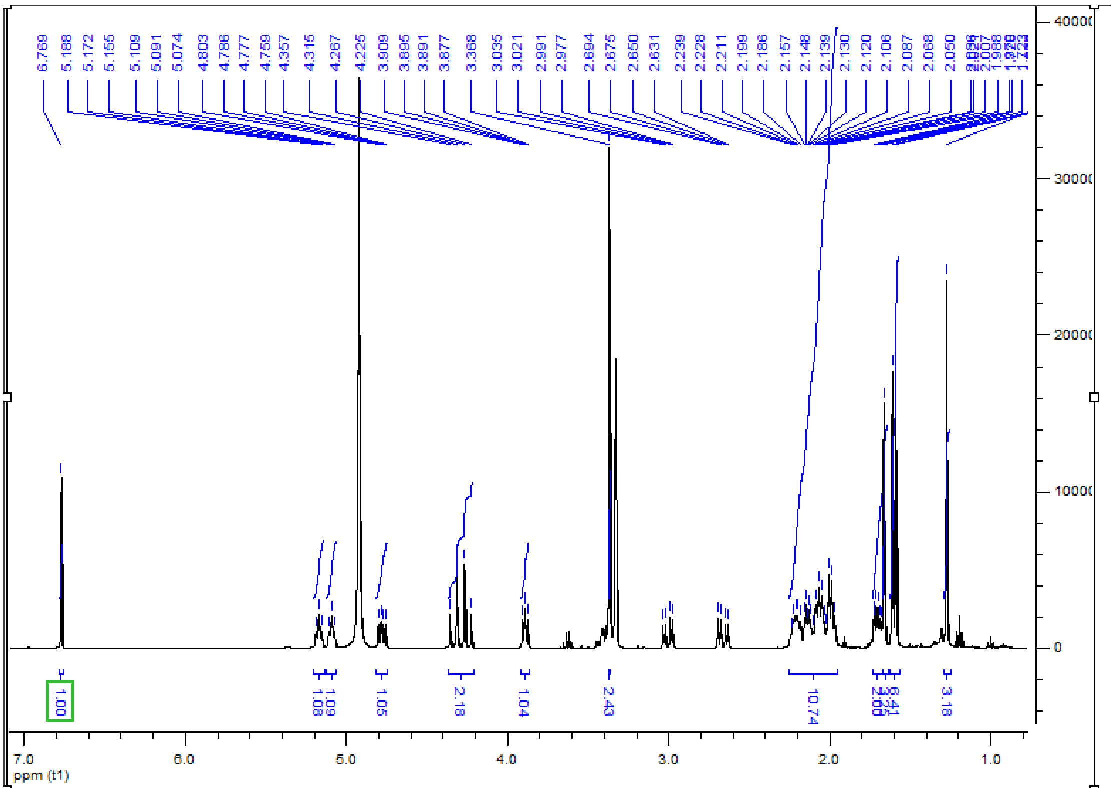


**Figure S5.** $^{1}$H NMR Spectrum of FGFC5 in MeOH-*d*4.

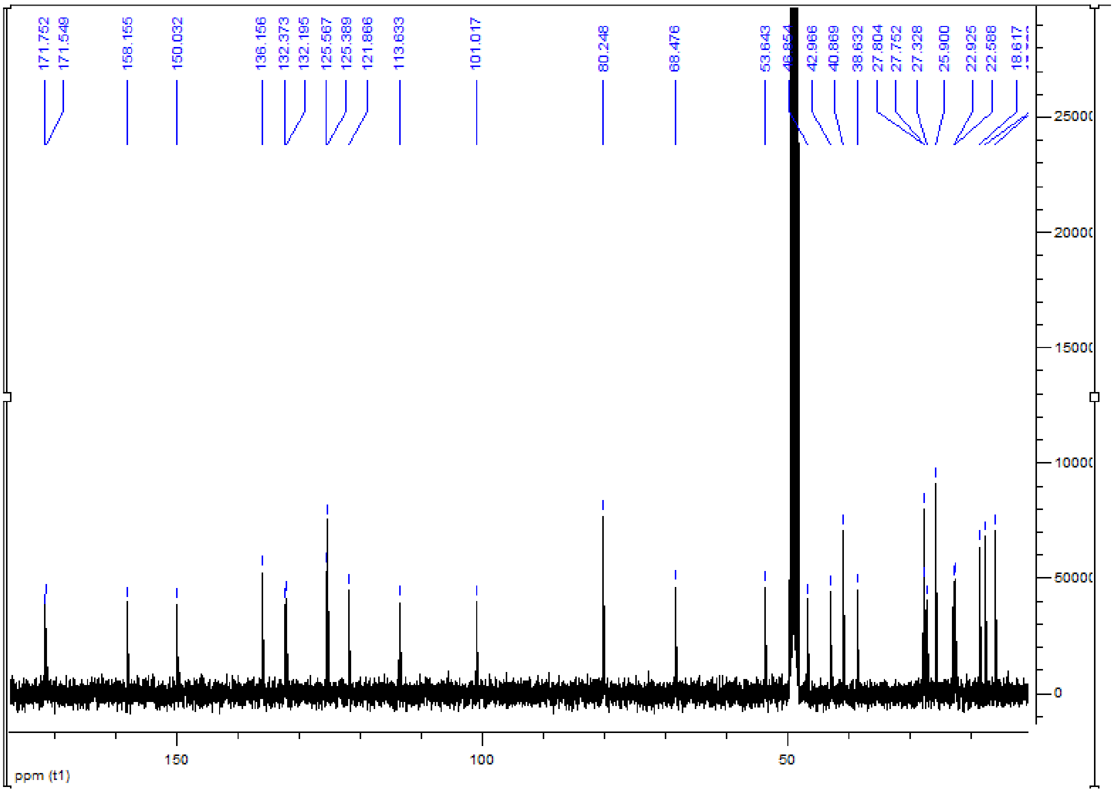


**Figure S6.** $^{13}C$ NMR Spectrum of FGFC5 in MeOH-d4.

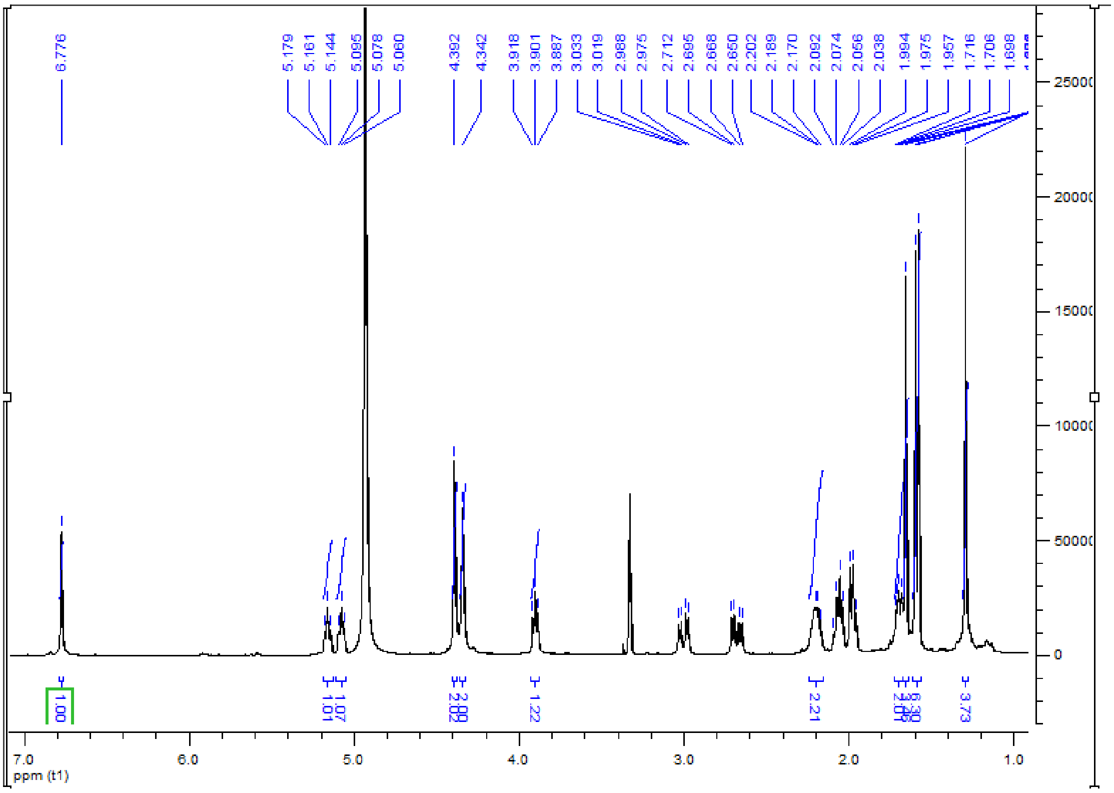


**Figure S7.** $^{1}H$ NMR Spectrum of FGFC6 in MeOH-$d_4$.

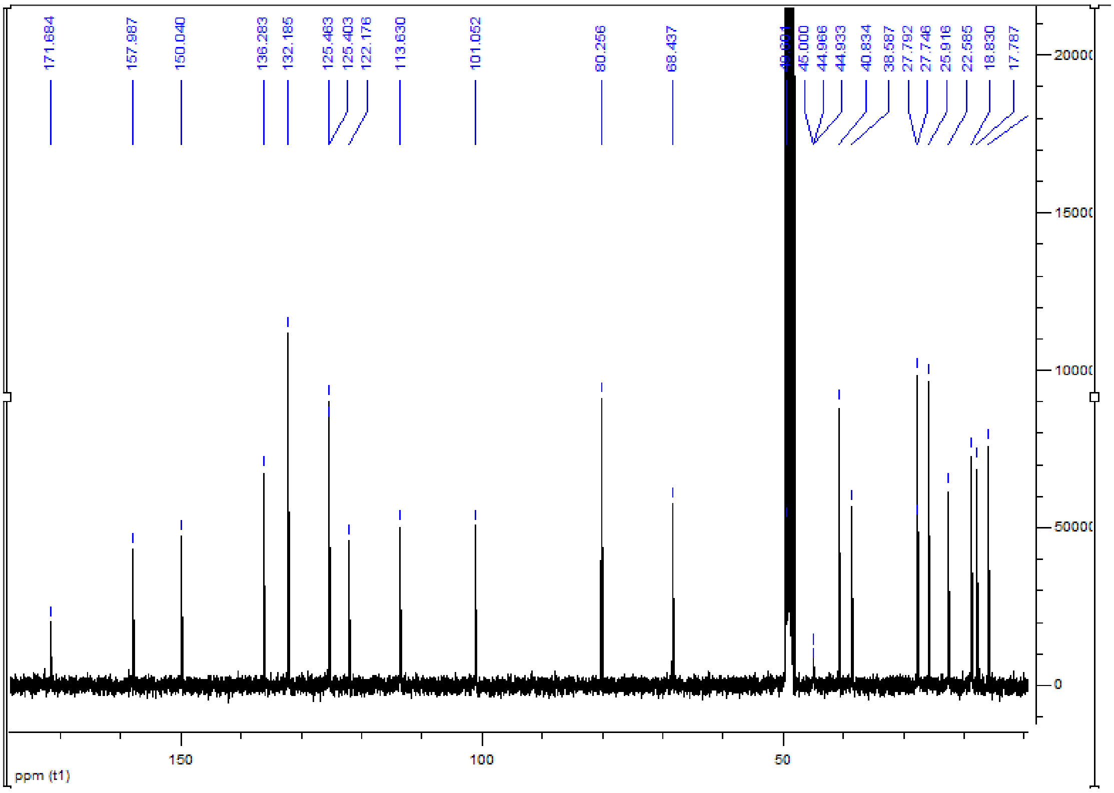


**Figure S8.** $^{13}C$ NMR Spectrum of FGFC6 in MeOH-$d_4$.

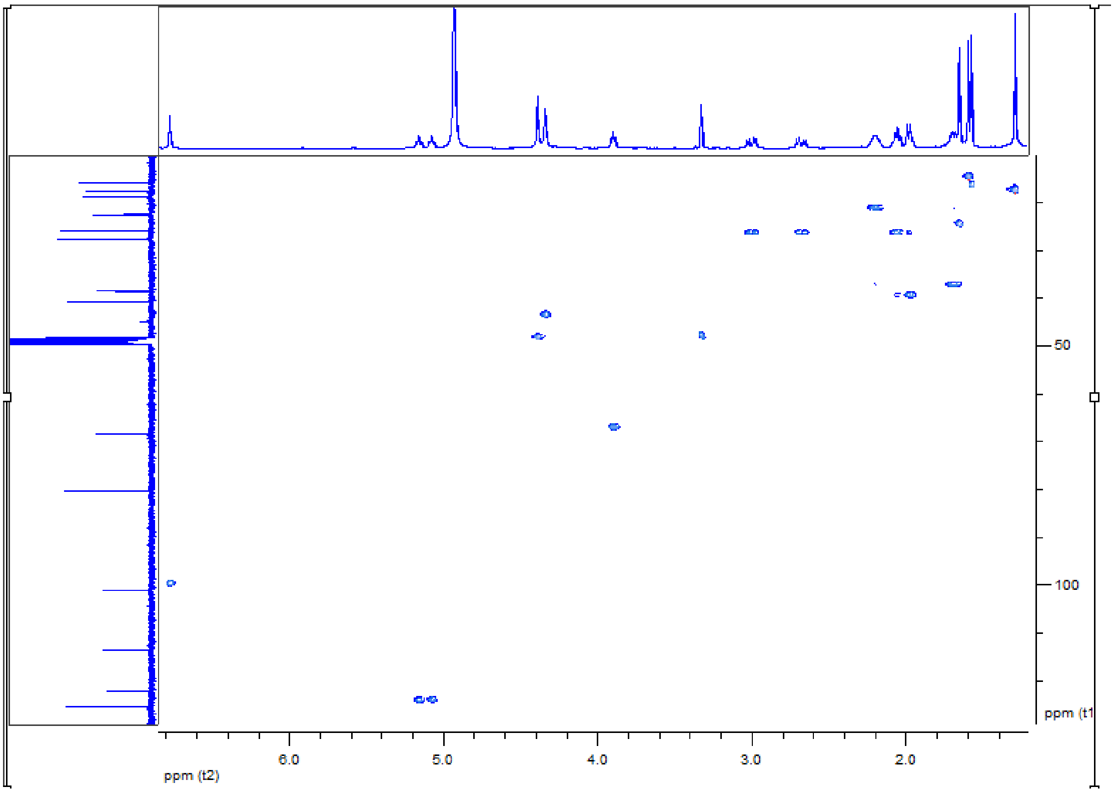


**Figure S9.** HSQC Spectrum of FGFC6 in MeOH-$d_4$.

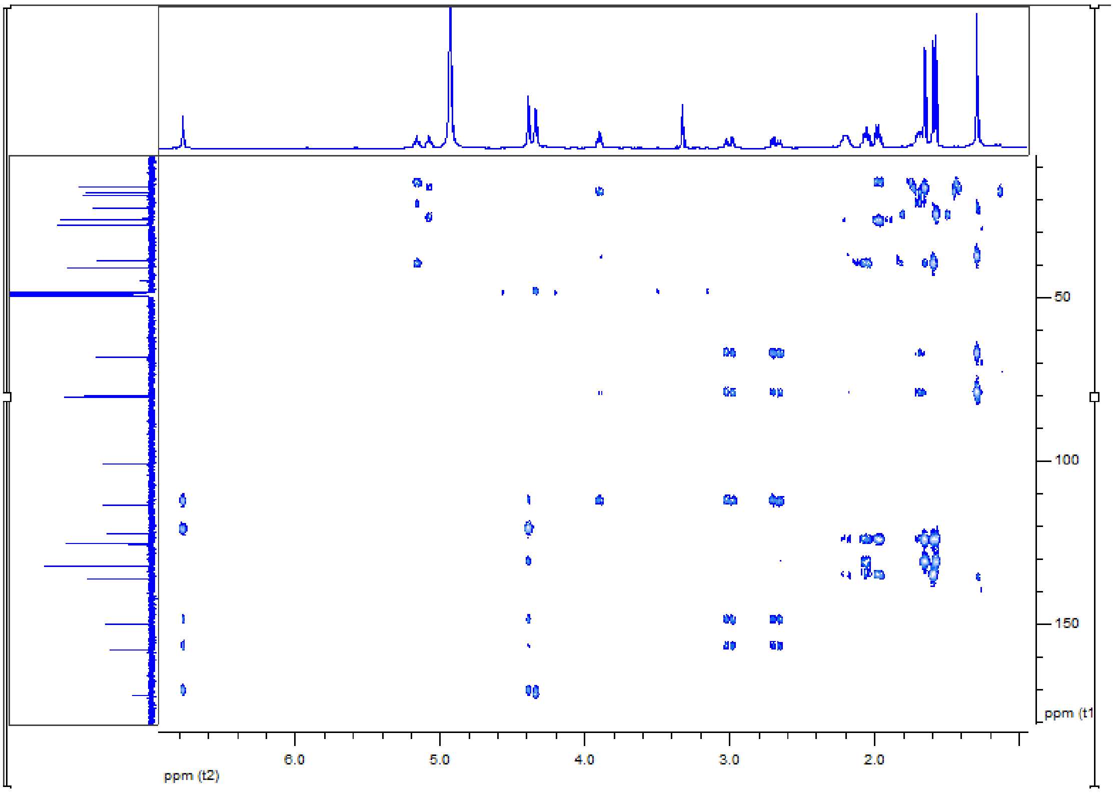


**Figure S10.** HMBC Spectrum of FGFC6 in MeOH-$d_4$.

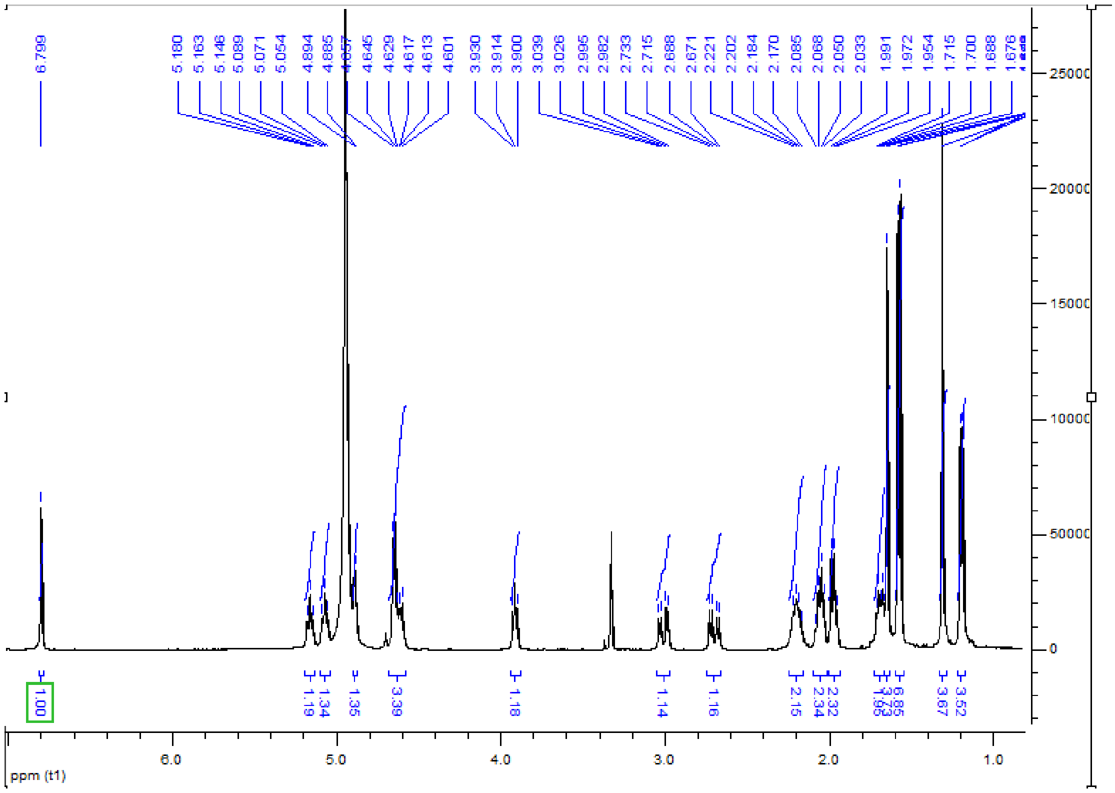


**Figure S11.** $^{1}$H NMR Spectrum of FGFC7 in MeOH-$d_4$.

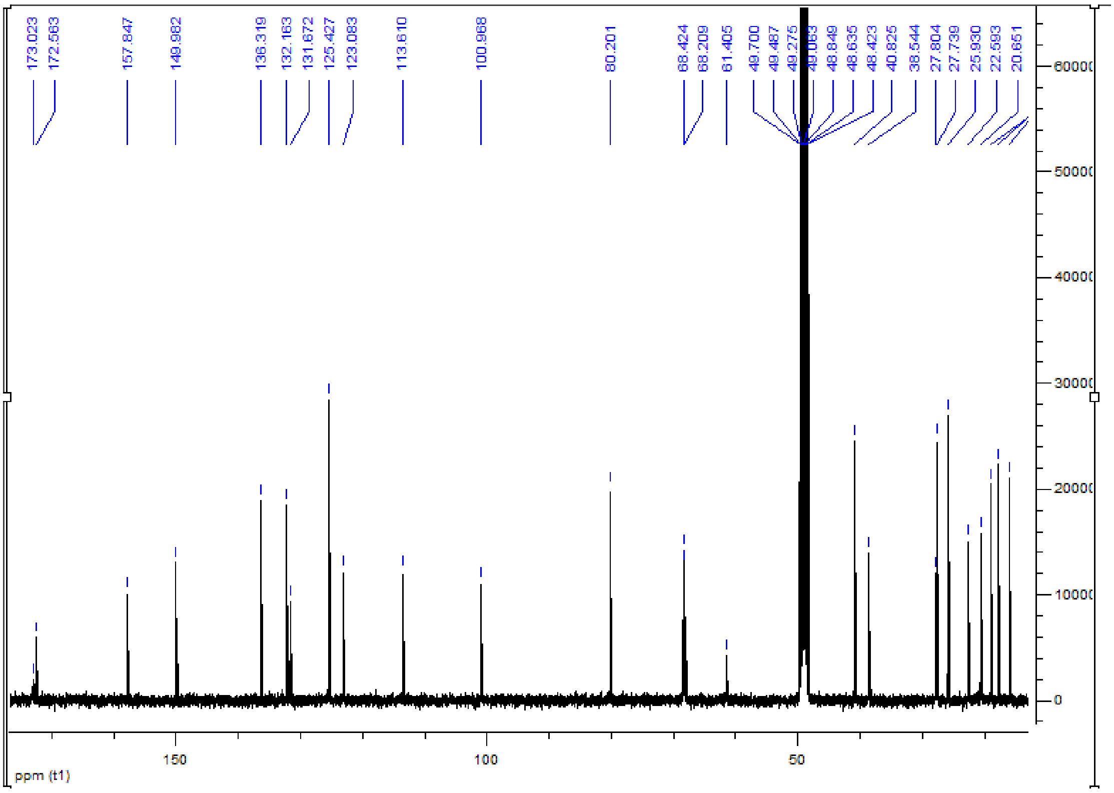


**Figure S12.** $^{13}C$ NMR Spectrum of FGFC7 in MeOH-$d_4$.

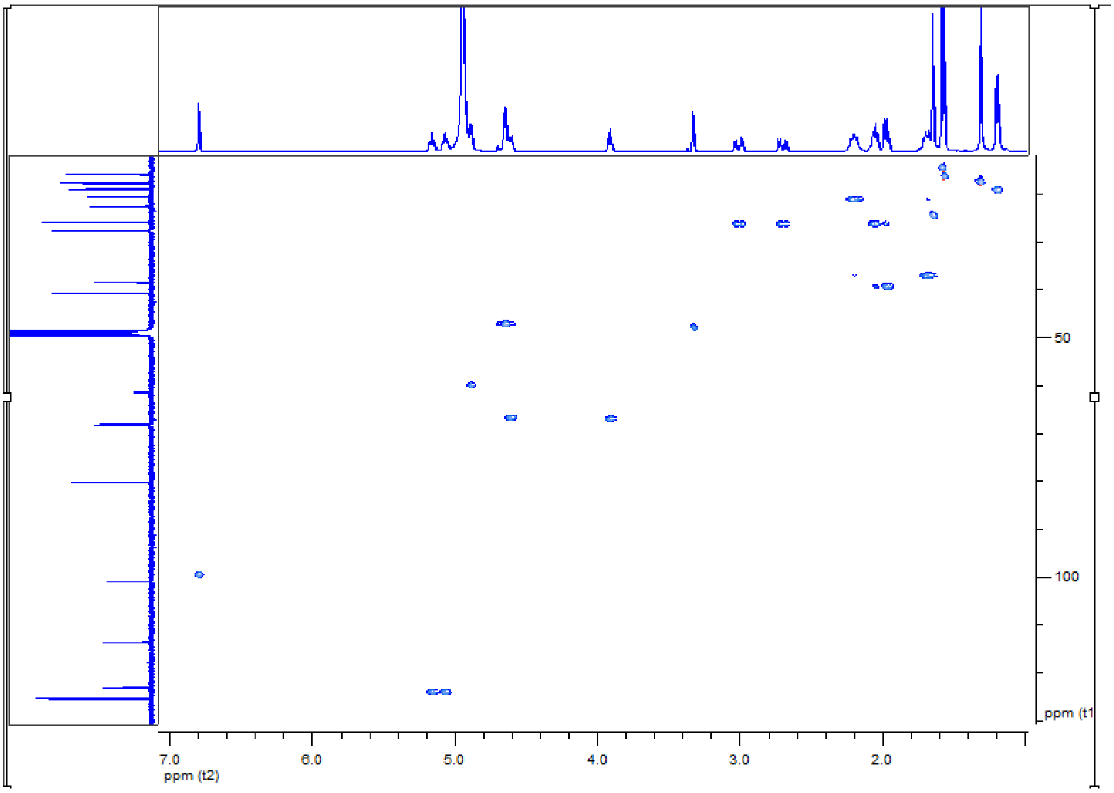


**Figure S13.** HSQC Spectrum of FGFC7 in MeOH-$d_4$.

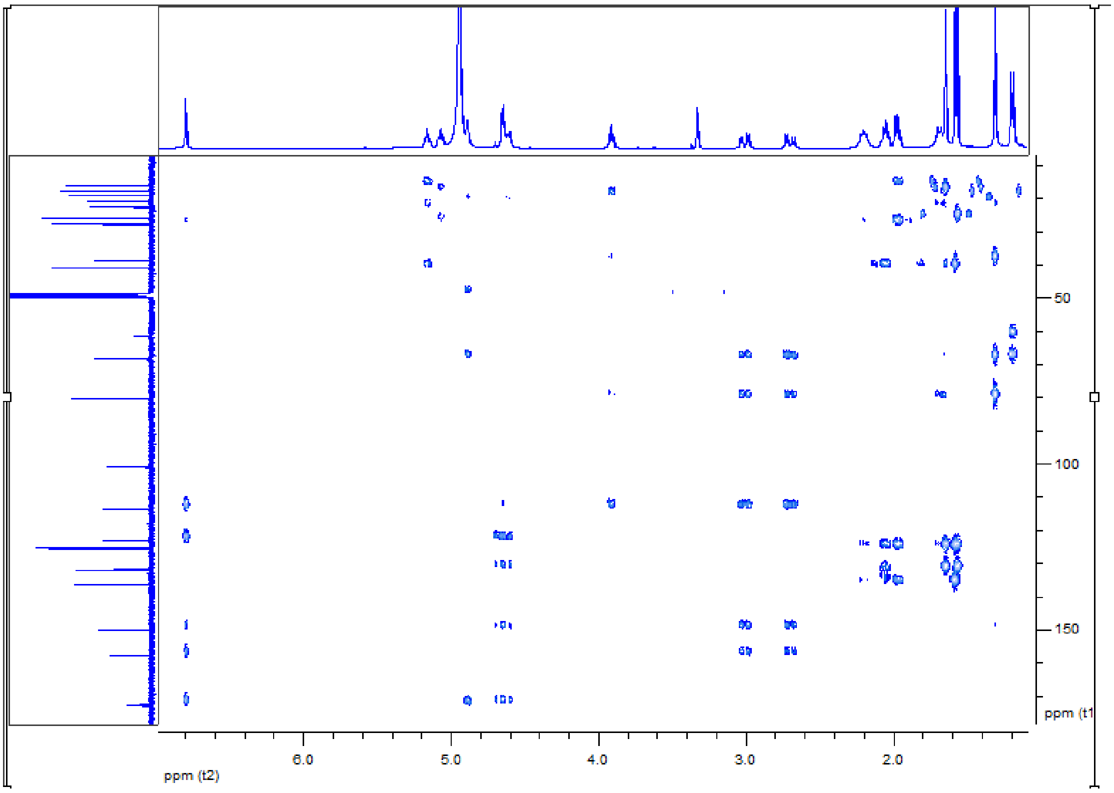


**Figure S14.** HMBC Spectrum of FGFC7 in MeOH-$d_4$.
